# The Current State of Targeted Agents in Rectal Cancer

**DOI:** 10.1155/2012/406830

**Published:** 2012-05-17

**Authors:** Dae Dong Kim, Cathy Eng

**Affiliations:** ^1^Department of Surgery, Catholic University of Daegu, 3056-6 Daemyung-4 Dong, Nam-Gu, Daegu 705-718, Republic of Korea; ^2^Department of Gastrointestinal Medical Oncology, MD Anderson Cancer Center, The University of Texas, 1515 Holcombe Boulevard, Box 0426, Houston, TX 77030, USA

## Abstract

Targeted biologic agents have an established role in treating metastatic colorectal cancer (CRC), and the integration of targeted therapies into the treatment of CRC has resulted in significant improvements in outcomes. Rapidly growing insight into the molecular biology of CRC, as well as recent developments in gene sequencing and molecular diagnostics, has led to high expectations for the identification of molecular markers to be used in personalized treatment regimens. The mechanisms of action and toxicities of targeted therapies differ from those of traditional cytotoxic chemotherapy. Targeted therapy has raised new insight about the possibility of tailoring treatment to an individual's disease, the assessment of drug effectiveness and toxicity, and the economics of cancer care. This paper covers the last decade of clinical trials that have explored the toxicity and efficacy of targeted agents in locally advanced and metastatic CRC and how their role may benefit patients with rectal cancer. Future efforts should include prospective studies of these agents in biomarker-defined subpopulations, as well as studies of novel agents that target angiogenesis, tumor-stromal interaction, and the cell signaling pathways implicated in rectal cancer.

## 1. Introduction

Over the past 30 years, the management of rectal cancer has undergone many significant changes. Until the 1980s, surgery was the mainstay of therapy for patients with rectal cancer confined to the bowel and regional lymph nodes [1]. However, local recurrence occurred in approximately 25% to 50% of patients with T3 or lymph node-positive rectal cancer [2]. These local failures, as well as distant metastases, were a serious problem in locally advanced rectal cancer (LARC). 

To reduce these high failure rates, multiple trials evaluated different strategies of adjuvant radiation and 5-fluorouracil- (5-FU-) based chemotherapy [1, 3, 4]. Trial results demonstrated postoperative adjuvant chemoradiotherapy improved local control and survival compared with surgery alone, leading to the routine integration of adjuvant combined modality therapy into standard practice. At the same time, total mesorectal excision (TME) was introduced and further decreased local failure rates to less than 10% [5]. Subsequently, the landmark trial conducted by the German Group established superior local control, reduced treatment-related toxicity, and an improved sphincter preservation rate with neoadjuvant chemoradiotherapy compared with adjuvant 5-FU-based chemoradiation [6].

Today, although not proven to provide survival advantages (except in the pivotal Swedish trial), preoperative chemoradiotherapy with concurrent infusional 5-FU and more recently the oral fluoropyrimidine, capecitabine, followed by TME has become the standard of care for patients with T3 or lymph-node-positive rectal cancer, especially in tumors of the mid- and lower rectum [7, 8]. The use of targeted agents in patients with advanced colorectal cancer has led to further improvements in disease-free (DFS) and overall survival (OS), and further investigation in various settings is underway [9–12]. These “targeted” agents are now being studied in the treatment of rectal cancer and are discussed below.

## 2. Targeted Agents

Targeted therapies block the growth of cancer cells by interfering with specific targeted molecules needed for carcinogenesis and tumor growth [13]. Targeted cancer therapies may also be more effective by being potentially less harmful to normal cells. Two main categories of targeted therapy exist: small molecules (-nib) and monoclonal antibodies (-mab), both of which can be further subdivided as either signal transduction pathway inhibitors (imatinib mesylate, trastuzumab, cetuximab) or angiogenesis inhibitors (bevacizumab, sunitinib).

Increasing knowledge of tumor growth and dissemination pathways has turned more attention to the use of targeted agents coupled with chemotherapy in the treatment of metastatic colorectal cancer (mCRC). For these patients, phase III trials have shown improved disease-free and overall survival rates using epidermal growth factor receptor (EGFR) and vascular endothelial growth factor (VEGF) inhibitors when combined with conventional chemotherapy [9–12]. In this paper, we have reviewed VEGF and EGFR receptor inhibitors selectively and how their use may or may not be beneficial in the setting of rectal cancer as a radiosensitizer or in the adjuvant setting of rectal cancer. The majority of novel trials discussed are in phase II development and are presented here due to their potential benefit in rectal cancer.

### 2.1. VEGF Receptor Inhibitors

Bevacizumab is a humanized monoclonal antibody that targets the vascular endothelial growth factor (VEGF), particularly VEGF-A, a ligand with a key role in angiogenesis. Angiogenesis is required for tumor growth and malignant progression, and VEGF is a crucial regulator of this process. Indeed, high VEGF expression has been linked to a statistically higher risk of local recurrence and metastasis [18]. Thus, the inhibition of VEGF is a logical target for the treatment of patients with CRC. In addition, anti-VEGF antibodies enhance the capacity of radiotherapy to reduce tumor vascular density and interstitial fluid pressure (IFP) in xenografts [19].

These findings taken together support what is known as the vascular normalization hypothesis [20]. According to this hypothesis, an excess of proangiogenic factors within tumors leads to functionally and structurally abnormal vasculature that promotes increased IFP, a known barrier to drug delivery to tumors, and impaired delivery of oxygen and macromolecules, a known barrier to the effective radiation therapy [20–22].

One theory is that by “normalizing” this abnormal vasculature, transient antiangiogenic therapy reduces IFP and thereby increases the concentration of oxygen and penetration of cytotoxics, improving the overall effectiveness of combined modality therapy [20]. In the same study, a variety of plasma and circulating cell biomarkers were measured. Both VEGF and placenta-derived growth factor (PIGF) were found to be significantly elevated by bevacizumab alone and by combination therapy. These molecules may prove to be potential biomarkers for anti-VEGF therapy, as increases of pretreatment soluble VEGF receptor-1 (s-VEGFR1) and PIGF levels have also been associated with poor pathologic tumor downstaging after preoperative chemoradiation [29, 30].

### 2.2. EGFR Receptor Inhibitors

The epidermal growth factor receptor (EGFR) is a cell surface 170,000 daltons tyrosine kinase transmembrane receptor of the ErbB family, whose members play a critical role in oncogenesis [31]. In particular, EGFR has been shown to participate in the progression of CRC, as it is essential for tumor growth and division [32]. Some CRCs have been shown to overexpress EGFR, and overexpression of EGFR is associated with poorer prognosis [33, 34], and with resistance to radiation therapy. EGFR has, therefore, become an attractive target for therapy, and two different classes of biologic agents have been evaluated: the EGFR monoclonal antibodies (cetuximab and panitumumab) and the small-molecule tyrosine kinase inhibitors (gefitinib and erlotinib).

The efficacy of both cetuximab and panitumumab has been clearly demonstrated to depend upon the KRAS mutation status. Multiple analyses have demonstrated that responses to either cetuximab or panitumumab occur exclusively within the 60–70% of patients without activating mutations in codon 12 and 13 of KRAS [23, 24, 28, 35]. The activating V600E BRAF mutation is present in an additional 10% of patients, and it may be predictive of a lack of response to anti-EGFR therapy [36] in addition to a clear poor prognostic factor. The anti-EGFR monoclonal antibodies and their predictive biomarkers have taken CRC treatment another step closer to personalized therapy. However, the recent results of the large UK COIN study and a Belgian study have not confirmed a benefit in terms of PFS or OS from the addition of cetuximab to oxaliplatin-based chemotherapy in wild-type KRAS patients versus 5-FU and oxaliplatin alone [37, 38]. Therefore, more thoughtfully designed studies about potential negative predictive factors such as KRAS, BRAF, NRAS, PIK3CA mutations, and EGFR gene copy numbers would be beneficial.

## 3. Clinical Uses of Targeted Agents

### 3.1. Metastatic CRC

Bevacizumab, cetuximab, and panitumumab have been proven to be effective in different combined chemotherapy treatment settings for metastatic colorectal cancer and are briefly described here (Tables 1 and 2) [9–12].

#### 3.1.1. Bevacizumab

Bevacizumab, a monoclonal antibody against the vascular endothelial growth factor ligand (VEGF-A), has demonstrated efficacy with significant improvement in progression-free survival (PFS) and OS in patients with metastatic colorectal cancer [9, 16, 39]. Results from a pivotal phase III trial of bevacizumab (5 mg/kg) showed a significantly greater OS, PFS, and response rate (RR) when used in combination with irinotecan, bolus 5-FU, and leucovorin (IFL), in 813 patients with previously untreated colorectal cancer [9]. The median OS was 20.3 months in the patients treated with bevacizumab, compared to 15.6 months with the placebo-containing regimen (*P* < .001), and response rates were 44.8% and 34.8%, respectively (*P* = .004). Also of note was the phase III Bevacizumab plus Irinotecan in Colorectal Cancer (BICC)-C trial, consisting of two-arms: FOLFIRI(infusional FL) plus bevacizumab, versus mIFL (Bolus FL) plus bevacizumab. The median OS with the addition of bevacizumab was longer with FOLFIRI than with mIFL (28.0 versus 19.2 months; *P* = .037) [40, 41]. These findings led to the U.S./European Union approval of bevacizumab as a first-line-therapy component for mCRC with any 5-FU- based therapy.

The most commonly used bevacizumab-based first-line treatment in the USA continues to be FOLFOX plus bevacizumab. It was presumed that the benefit of adding bevacizumab to FOLFOX would be similar to that demonstrated with the IFL regimen, and that the addition of bevacizumab to FU-based combination chemotherapy would result in a significant and clinically meaningful improvement in survival among patients with mCRC [39]. Except in cases of major contraindications for bevacizumab, such as severe vascular disease or prior arterial thrombotic events, bevacizumab can be integrated with first-line chemotherapy in patients with metastatic CRC. In the event that a bevacizumab-naïve patient fails first-line chemotherapy, bevacizumab may be considered as a second-line treatment. In fact, the Bevacizumab Regimens: Investigation of Treatment Effects and Safety (BRITE) observational study confirmed that a notable OS benefit was demonstrated in those patients who continued bevacizumab therapy (5 mg/kg every 2 weeks) in combination with chemotherapy, after disease progression following a bevacizumab-based regimen (median OS, 31.8 months) [42]. The ARIES study validated the findings of BRiTE and reported a median OS of 24.7 months [43]. Furthermore, a recent randomized phase III European trial (TML) suggests continuation of bevacizumab improves OS with final results to be reported at a later date [44].

#### 3.1.2. Cetuximab

Cetuximab plus irinotecan is the standard treatment in irinotecan-refractory patients based on pivotal data from the Bowel Oncology with Cetuximab Antibody (BOND) trial, which established this as an effective regimen regardless of prior treatment history [10]. This trial confirmed the activity of cetuximab with response rates of 22.9% and 10.8% for combination therapy and monotherapy, respectively. In addition,there was a significantly longer time to tumor progression in favor of the combination arm (4.1 versus 1.5 months).

In a subsequent retrospective analysis of patients with KRAS wild-type tumors, there was an OS benefit for patients in favor of cetuximab (9.5 versus 4.8 months) [28]. Given cetuximab's demonstrated efficacy in the chemotherapy treatment-resistant setting, several studies were undertaken to evaluate its efficacy in the treatment-naive setting. The randomized, phase III CRYSTAL trial evaluated 1198 patients treated with FOLFIRI chemotherapy either with or without cetuximab, and noted improvement in PFS for the cetuximab-treated group [23]. However, it was also noted that no OS benefit was associated with the cetuximab-treated arm, potentially due to the fact that there was a difference in subsequent poststudy anti-EGFR therapy (23% in the FOLFIRI arm received subsequent cetuximab therapy, compared to 5.2% in the FOLFIRI + cetuximab arm) [49]. Retrospective tissue analysis on the CRYSTAL study revealed that the benefit from cetuximab was restricted to those patients with KRAS wild-type tumors and that it improved the PFS to 9.9 months compared to 8.4 months in the control arm. Subsequent analysis of the 666 patients with KRAS wild-type tumors who were enrolled has shown an OS improvement from 20 months in the control arm to 23.5 months in the cetuximab arm [50].

The randomized Phase II OPUS trial demonstrated similar findings when cetuximab was used with oxaliplatin-based chemotherapy; cetuximab, when combined with FOLFOX-4 chemotherapy, led to an improvement in PFS, when compared to those treated with FOLFOX-4 chemotherapy alone [51]. Similarly, the benefit derived from this therapy was limited to those patients with KRAS wild-type tumors (7.7 versus 7.2 months). However, these results have not been replicated in the phase III MRC-COIN study, where cetuximab was added to FOLFOX or CapeOX (capecitabine/oxaliplatin) in the first-line setting; those treated with capecitabine-based therapy fared worse [37]. Based on currently available data, it appears to be advantageous when cetuximab is added to irinotecan-based regimens while the advantage of combination with oxaliplatin remains less certain [26, 52]. Cetuximab can lead cancer cells to G1 or G2/M cell cycle arrest and upregulate several genes involved in proliferation (PIK31, CGREF1, and PLAGL1) with a reduction in Ki-67 [38]. But if only a small proportion of cells arrest in G0/G1 or G2/M, slowing down the cell cycle time may actually increase the amount of time available for DNA repair prior to mitosis, and thus could increase resistance to chemoradiation.

#### 3.1.3. Panitumumab

Two phase III trials have determined the benefit of panitumumab in combination with chemotherapy relative to chemotherapy alone. The final reported results were selected for KRAS wild-type status as a predictive marker for anti-EGFR therapy in both studies. The Panitumumab Randomized Trial in Combination with Chemotherapy for Metastatic Colorectal Cancer to Determine Efficacy (PRIME) was a phase III randomized trial of FOLFOX with or without panitumumab in previously untreated patients [53]. The PFS time was longer in the panitumumab arm (9.6 versus 8.0 months, *P* = .02). The role of panitumumab was also investigated in combination with FOLFIRI for second-line treatment [54]. The primary endpoint of a PFS difference (5.9 versus 3.9 months; *P* = .004) was fulfilled with the addition of panitumumab but the OS endpoint was not met.

### 3.2. Adjuvant Setting

#### 3.2.1. Bevacizumab

In the adjuvant treatment of colon cancer, two phase III trials did not demonstrate any benefit from the addition of bevacizumab to standard oxaliplatin-based chemotherapy in stage II/III colon cancer [55, 56]. From the results of the NSABP C-08 trial, in which bevacizumab in combination with FOLFOX6 did not improve DFS in the adjuvant setting in patients with stage II/III colon cancer, it seems that bevacizumab's efficacy may be maximal in a setting of more advanced disease [1]. The AVANT (Avastin adjuvant) study compared FOLFOX4 versus FOLFOX4 with bevacizumab versus XELOX with bevacizumab in 3,451 patients with stage II or III colon cancer. The study did not meet its primary objectives, and survival in the bevacizumab arms was inferior to the chemotherapy-alone arm [57].

#### 3.2.2. Cetuximab

Two adjuvant trials have evaluated the potential benefit of cetuximab in combination with FOLFOX in a KRAS wild-type population: NO147 and PETACC8. There was no improvement in DFS or OS with the addition of cetuximab to FOLFOX. The NO147 trial compared 12 cycles of the modified FOLFOX6 to the same regimen with cetuximab [58, 59]. The 3-year DFS was even better with FOLFOX alone: 75.8% versus 72.3% for FOLFOX plus cetuximab. There was also a trend for 3-year OS inferiority: 87.8% with FOLFOX alone versus 83.9% for FOLFOX plus cetuximab. The subset of 717 patients with KRAS-mutated tumors did poorly with cetuximab. These observations raise questions about the manner of interaction between targeted therapies and chemotherapies and the mechanisms of inducing chemoresistance in some patients. The PETACC8 trial aims to increase the DFS in wild-type KRAS tumor patients. The trial has completed enrollment with final results to be presented at a later date.

Thus far, we have provided a general overview of the currently FDA approved role for targeted agents in metastatic colorectal cancer as well as the investigational status of targeted agents in locally advanced colon cancer. Overall, of the targeted agents discussed, many of them have been analyzed to a much smaller extent in rectal cancer versus that of colon cancer in phase I/II clinical trials.

## 4. Locally Advanced Rectal Cancer

Overall, challenges remain in improving the treatment of locally advanced rectal cancer. Recent data from 3 phase III trials including NSABP R-04, STAR, and ACCORD 12 [60–62] have failed to provide a definitive role for oxaliplatin as a radiation sensitizer. Targeted agents in combination with fluorouracil-based treatment continue to remain an area of investigation.

### 4.1. Neoadjuvant Chemoradiation

Randomised phase III trials of neoadjuvant preoperative chemoradiation (CRT) in resectable rectal cancer show that the addition of 5-FU to preoperative radiation increases the pathologic complete remission (pCR) rate over radiotherapy alone and improves loco-regional control but has not extended DFS or OS [6, 63–65]. Several small phase I/II studies have since been created in the hopes of improving the pCR rate further by using combined chemotherapy with cytotoxic chemotherapy or targeted agents. It should be noted that pCR remains a common endpoint for many of current and prior trials in development. To date, none of the targeted agents have proceeded to phase III development as a radiation sensitizer with only one proceeding to the adjuvant setting of rectal cancer.

#### 4.1.1. Bevacizumab

Given the data supporting the efficacy of bevacizumab therapy in metastatic colorectal cancer [17], it was postulated that combining bevacizumab with chemoradiation may increase antitumour efficacy by maximizing inhibition of the VEGF pathway. In an early trial of bevacizumab plus infusional 5-FU and radiation in stage II/III rectal cancer, bevacizumab administered as a single agent 14 days prior to chemoradiation induced normalization of tumour vasculature and reduced interstitial fluid pressure [66]. Willett et al. first reported on a phase I trial of bevacizumab in combination with preoperative 5-FU and radiation. Surgery was scheduled 7–9 weeks later. A pCR rate of 16% was reported, and an additional 72% of patients had only microscopic foci remaining after treatment in their phase I/II study of patients with T3/T4 tumors [29]. Crane et al. reported results of a single institution phase II trial of neoadjuvant bevacizumab, capecitabine, and radiation in 25 patients with locally advanced rectal cancer [47]. The pCR rate was 32%, with an additional 24% of patients having near-complete responses (less than 10% viable tumor cells). Toxicity was generally mild, although wound dehiscence was seen in 12% of patients (Table 3). 

It should be noted that bevacizumab was to be evaluated in the adjuvant setting following definitive chemoradiation therapy in locally advanced rectal carcinoma patients (ECOG 5204). This was a randomized phase III trial of adjuvant FOLFOX +/− bevacizumab. However, this study closed prematurely due to slow patient accrual but would have closed eventually given the results of NSABP C-08.

#### 4.1.2. Cetuximab

EGFR is also a rational target in combination with radiotherapy. Data from a variety of experimental models and human tumors suggest that EGFR signaling promotes resistance to radiotherapy by activating cell survival signals through Akt [73, 74]. Moreover, retrospective analyses demonstrated shorter DFS and smaller pCR rates in patients with rectal cancers expressing EGFR who were being treated with neoadjuvant radiotherapy [75, 76]. These data suggest the possibility of enhancing radiosensitivity with EGFR-directed therapy.

A landmark randomised phase III study in patients with locally advanced head and neck cancer showed that cetuximab in combination with radical radiotherapy significantly improved overall survival [77] compared to radiation alone [78]. Many mechanisms for this advantage have been proposed, including inhibition of repopulation during the latter phase of radiotherapy [79, 80]. Chung et al. treated 20 patients with ultrasound-staged (u)T3 to 4, clinical T4, or locally recurrent rectal cancer with weekly cetuximab and 5-FU during 5.5 weeks of pelvic radiotherapy, followed by an additional 4 weeks of cetuximab [81]. Patients underwent surgical resection 1 to 3 weeks after the completion of therapy. Cetuximab in conjunction with 5-FU and radiotherapy was feasible without synergistic or unexpected toxicities; the pCR rate was 12%. According to the meta-analysis of 13 reports, the addition of cetuximab to fluoropyrimidine-based CRT schedules suggest an overall pooled pCR of 9.1%, compared with an overall pCR rate of 13.5% seen with fluoropyrimidine-based chemoradiation schedules in a recent review (Table 4) [82]. The pCR rates have been disappointingly low, perhaps because anti-EGFR therapy is most effective in inhibiting the accelerated repopulation observed after the higher doses of radiotherapy used in patients with head and neck cancer [47].

 Alternatively, cetuximab-induced inhibition of mitogenic signaling through the extracellular signal-regulated kinase (Erk) pathway may mitigate the radiosensitizing effect of S phase-specific chemotherapy. Cetuximab can lead to G1 or G2/M cell cycle arrest, and if only a small proportion of cells within the tumour are affected, this decrease in proliferation could impact the chance of achieving a pCR [38]. This process might also affect oxaliplatin, which is mainly active in S phase but would be less likely to affect irinotecan. Thus, preclinical data suggests that the sequencing of chemotherapy may have some significance but given the prolonged half-life of cetuximab, it may not necessarily apply to the clinical setting [83]. In light of this hypothesis, a phase I/II study (XERXES) created to compare a schedule of capecitabine-based chemoradiation with a cetuximab sandwich approach amended their protocol [84].

#### 4.1.3. Panitumumab

Panitumumab was administered before the start of CRT, and every 2 weeks in combination with 5FU-oxaliplatin with concurrent RT(StarPan/STAR-02 study) [85]. Rate of pCR was 21.1% and pathological downstaging occurred in 57.9% of the patients. Higher pCR rate in comparison with the results of previous neoadjuvant rectal cancer trials with antiepidermal growth factor receptor monoclonal antibodies supports further studies necessary to understand the possibility of optimal regimens and sequences with CRT.

#### 4.1.4. Tyrosine Kinase Inhibitors of the EGFR

Though it would appear to be intuitive that a tyrosine kinase inhibitor of the EGFR receptor would be of potential benefit, early data for their role in mCRC has not been defined and was determined to be more toxic [67, 86]. However, the small molecule tyrosine kinase inhibitors have radiosensitization properties. A phase I trial of a combination of erlotinib and bevacizumab with preoperative chemoradiotherapy of capecitabine demonstrated an impressive pCR(44%) [87].

Similar preliminary results were also reported of a phase I/II trial of bevacizumab and erlotinib in combination with 5-FU and pelvic radiotherapy for patients with clinical T3/4 rectal cancer [88]. No dose-limiting toxicities were reported and erlotinib at a dose of 100 mg was chosen as the MTD. At the time of last followup, the reported pCR was 47% and there were no local recurrences reported in patients who completed study therapy. Thus, based on these phase I/II trials, bevacizumab and erlotinib in combination with 5-FU or capecitabine and radiotherapy appear to be well tolerated and a potentially active preoperative regimen for patients with LARC.

### 4.2. Induction Chemotherapy

While modern rectal cancer trials with trimodality therapy have reported locoregional recurrence rates of less than 10%, especially with TME, systemic treatment can be delayed for up to 3-4 months following the original diagnosis [89, 90]. The problem, therefore, remains the persistent high rate of distant metastasis (30–35%) in this disease [91, 92]. Reasons for this higher rate of distant failure may be complex. One such reason, the biological response of tumors to radiation, may provide explanation in that tumors that are not completely eradicated undergo accelerated repopulation [93].Thus, integrating more effective systemic therapy into combined modality programs is the challenge and induction chemotherapy has the advantage of earlier administration of systemic therapy and may improve distant control. Theoretically, tumour shrinkage with chemotherapy potentially allows improved tumour vascularity and improved oxygenation and higher intratumoural levels of cytotoxic drugs. These factors in turn may enhance tumour sensitivity to chemotherapy or radiation [94]. Therefore, newer generation chemotherapeutics as well as targeted agents (cetuximab, bevacizumab) have been incorporated into phase I-II studies [95, 96].

Interestingly, early results from the phase III ACCORD 12/0405-Prodige 2 and STAR trial did not confirm a significant improvement of the primary endpoint, pCR rate, with the addition of oxaliplatin to preoperative 5-FU-based CRT [91, 92]. However, induction-capecitabine-based CT prior to CRT reported eventual pCR rates as high as 24% [97, 98]. In a randomized comparison of induction versus adjuvant capecitabine plus oxaliplatin, no difference in clinical outcomes was observed between the two treatment regimens, although the induction regimen resulted in a more favorable safety profile [99]. To date, the 3-year DFS and OS remain unchanged versus the control arm [100].

In the AVACROSS study, treatment consisted of bevacizumab and XELOX, followed by concomitant radiotherapy plus bevacizumab and capecitabine. pCR was achieved in 36% of the patients but the rate of postsurgical complications and cardiac toxicity was not negligible. Most were associated with wound-healing complications with 24% of patients requiring further surgery; note that patients with a previous history of stable angina, arrhythmia, and coronary syndrome should be excluded [101]. Emerging evidence from several phase I-II trials indicate that induction chemotherapy for rectal cancer patients is feasible and does not compromise CRT or surgery, but only an adequately powered phase III trial will answer the question definitively.

An interesting multinational randomised phase II study, EXPERT-C trial (NCT00383695), compares neoadjuvant therapy comprising oxaliplatin, capecitabine, and CRT with or without cetuximab in 164 patients. It was designed on the basis of earlier single-arm phase II study (EXPERT), in which patients received 12 weeks of neoadjuvant capecitabine and oxaliplatin followed by chemoradiotherapy with capecitabine, TME, and 12 weeks of postoperative adjuvant capecitabine [97]. Radiological response rate after neoadjuvant chemotherapy and chemoradiotherapy was 89% (93/105) with a reported pCR of 20%. Three-year PFS and OS were 68% and 83%, respectively. Early results of the EXPERT-C trial indicate that improved 3-yr OS in patients receiving the investigational arm with CRT was 96% compared with 81% among those who received neoadjuvant chemotherapy and chemoradiation alone [102]. In the KRAS wild-type group, there was no statistically significant difference in PFS (81 versus 80%) and pCR (7 versus 11%) [7]. Though a phase II trial, the results of EXPERT-C have potentially renewed interest of investigators for oxaliplatin and cetuximab in the treatment of rectal cancer; results will need to be validated in a phase III trial.

### 4.3. Novel Targeted Agents in Phase III Clinical Trials

Several novel agents are being studied in phase III trials for the treatment of metastatic colorectal cancer: aflibercept, ramucirumab, regorafenib, perifosine, and brivanib. Of these, aflibercept and ramucirumab are specific VEGF-directed therapies, whereas the remaining three are associated with approaches to intracellular signal blockade.

Aflibercept is a fully human, recombinant fusion protein that functions as a soluble decoy receptor for VEGF, with affinity for VEGF-A, VEGF-B, and placental growth factor [103]. The VELOUR trial is a randomized, placebo-controlled study investigating the combination of FOLFIRI plus aflibercept versus FOLFIRI in the second-line treatment of mCRC [104]. Ramucirumab is another human monoclonal antibody directed against VEGFR-2, which is considered to be the primary VEGFR mediating the process of tumor angiogenesis [105, 106]. It is currently in phase III development in 2nd-line metastatic colorectal cancer in combination with FOLFIRI [107]. 

Regorafenib is a diphenylurea oral multikinase inhibitor that targets a variety of kinases implicated in angiogenic and tumor growth-promoting pathways and has been investigated as a single-agent activity in the phase III CORRECT trial in refractory mCRC [108]. The final results of the CORRECT trial noted improved OS versus best supportive care alone (6.5 versus 5.0 mo, *P* < .0052) [109]. Perifosine is an oral alkylphospholipid that inhibits several key signal transduction pathways, including Akt, JNK, and NF-*κ*B. Inhibition of NF-*κ*B signaling by perifosine has been reported to restore 5-FU chemosensitivity [110, 111]. The Xeloda plus Perifosine Evaluation in Colorectal Cancer Treatment (X-PECT) trial with a target enrollment of 430 patients has recently completed accrual [112]. Perifosine has been evaluated preclinically in prostate and glioma as a radiation sensitizer [113, 114]. Brivanib is an oral receptor tyrosine kinase inhibitor that specifically inhibits the VEGF and fibroblast growth factor (FGF) signaling pathways [115, 116]. Recent phase III data failed to show a benefit in OS when brivanib was added to cetuximab in KRAS-WT metastatic CRC patients [117]. Brivanib continues to be investigated in combination in a single institutional trial with irinotecan in an enriched patient population with a focus on plasma fibroblast growth factor (pFGF) [118].

Currently, perifosine appears to be the only targeted agent in phase III development with some potential as a radiation sensitizer based on preclinical studies. Hence, though greater than 50 novel agents are being investigated in phase II trials, only a small percentage of these agents will proceed to phase III clinical trial development with a minority eventually investigated for their radiosensitization properties. Therefore, it is important to identify predictive biomarkers for chemotherapy and radiation sensitivity, a task that should involve close cooperation and discussion between basic scientists, clinical, and translational investigators.

## 5. Adverse Events Associated with Targeted Therapy

### 5.1. Bevacizumab

Bevacizumab is accompanied by a manageable safety profile. Early-phase II and pivotal-phase III studies in CRC utilizing bevacizumab have identified hypertension (11%–32%), bleeding (mainly epistaxis; 30%–53%), proteinuria (10%–38%), arterial thromboembolism (ATE) (1%–10%), gastrointestinal perforation (0.3%–2%), and wound healing (1.3%–3.7%) as bevacizumab-associated adverse events.

Hypertension is the most commonly reported associated toxicity of patients treated with bevacizumab [9, 42]. The mechanism of bevacizumab-related HTN is unclear and has been attributed to possible alterations in the nitric oxide signaling pathway and the endothelial dysfunction of the renin-angiotensin system [48, 119]. There are no clear guidelines for the management of hypertension in patients receiving bevacizumab, but patients who develop more than grade 2 HTN during bevacizumab therapy should be managed using standard antihypertensive therapy.

Incidence of grade 3/4 proteinuria in recent phase III trials with bevacizumab in metastatic CRC have been reported in less than 2% of patients [9, 42]. The mechanism of proteinuria is not fully understood but may be related to the effects of VEGF on the renal glomerular capillaries. It is advisable that patients with proteinuria of more than 2+ on a dipstick should have a 24-hour urine check for quantification of protein. If patients develop proteinuria of more than 2 g/24 hours, bevacizumab administration should be interrupted until proteinuria improves. Patients should be evaluated for ACE inhibitors or angiotensin-receptor blockers as initial treatment in the case of development of proteinuria with hypertension.

Bleeding from mucocutaneous membranes is common with bevacizumab and occurs in 20% to 40% of patients [120], the most significant type being gastrointestinal bleeding (6%) and the most common being self-limited epistaxis (46%). Note that the BRiTE study did not exclude patients on antiplatelet therapy or full-dose anticoagulation [121], so it is relatively safe in terms of risk of serious bleeding complications.

Thrombosis is a more potentially serious adverse event. In the pivotal phase III trial in patients with metastatic CRC evaluating the IFL regimen with and without bevacizumab, the incidence of all venous and ATE was 19.4% versus 16.2% in the nonbevacizumab arm [9]. It has been speculated that the blockade of the VEGF receptors leads to apoptosis of endothelial cells in the vasculature, thus exposing the subendothelial collagen and initiating a coagulation cascade resulting in an increased risk for thrombus formation [122]. Thus, the Food and Drug Administration (FDA) described that the rate of ATE is increased in patients with prior events of ATE or patients aged more than 65 years during bevacizumab therapy.

Other less common but serious reported toxicities may include gastrointestinal perforation (2%) and wound-healing complications. Patients who underwent surgery within 14 days of receiving bevacizumab are at a higher risk of developing wound healing complications [14]. Based on available data, bevacizumab should not be initiated within 30 days after surgery. Elective surgeries should be planned 6 to 8 weeks after last dose of bevacizumab, though chemotherapy alone can be continued until 2 to 3 weeks before surgery. Furthermore, it has been suggested that extended use of bevacizumab can increase the long-term risk of wound healing complications for up to 6 months after its cessation [55, 123].

The incidence of GI perforations may be slightly higher in patients with an intact primary tumor, prior adjuvant radiation therapy in rectal cancer, long-term nonsteroidal anti-inflammatory drug (NSAID) therapy (≥1 month of use), peptic ulcer disease, diverticulosis, and previous gastrointestinal surgery [9, 100, 124]. An extensive meta-analysis reported an incidence of GI perforation under bevacizumab treatment of <1%, resulting in a mortality of 22% [125]. Reversible posterior leukoencephalopathy syndrome (RPLS) has been reported in clinical studies (with an incidence of < 0.1%) and in postmarketing experience [126]. RPLS is a neurological disorder, which can present with headache, seizure, lethargy, confusion, blindness, and other visual and neurologic disturbances. Discontinuation of bevacizumab and aggressive management of hypertension is indicated in patients developing RPLS.

### 5.2. Cetuximab

The most frequently observed toxicity with the use of cetuximab is the development of a rash; all patients experience some degree of xerodermatitis. The majority will develop a nonscarring, noninfectious, acneiform rash most prominent on the malar aspect of the face, chest, and back; it is exacerbated by sunlight. But the severity of the rash has been correlated with improved response to cetuximab therapy [127]. In view of this finding, a randomized, Phase I-II study (the “EVEREST” trial) was developed, with the experimental arm consisting of dose escalation of cetuximab to rash development in those treated patients who failed to develop a grade 2 rash following initial exposure to cetuximab. While a higher radiographic response rate in the experimental arm of this trial (the cetuximab dose escalation to rash) was observed, this did not lead to an improvement in PFS or OS advantage [128].

Cetuximab has also been frequently associated with renal magnesium wasting leading to hypomagnesemia, with this occasionally becoming symptomatic [129, 130]. When symptomatic, repletion with enteral or parenteral magnesium is indicated; it is not clear that magnesium replacement is beneficial in an asymptomatic patient [131]. Paronychial inflammation and fissuring of the distal fingertips are a class effect of these agents. If left unaddressed, severe paronychial infections may develop. Instances of elevated cases of allergic hypersensitivity reactions have been reported regardless of the premedications provided [132]. The development of IgE antibodies was noted and varied depending on the geographic location with greater instances in the in the Southern USA.

### 5.3. Panitumumab

In contrast to cetuximab, which is a chimeric monoclonal antibody that may produce severe hypersensitivity reactions in some patients, panitumumab is a fully human monoclonal antibody and is less likely to result in allergic hypersensitivity reaction.

## 6. Pharmacoeconomic Considerations

Targeted therapy also introduces new economic considerations. Targeted therapy is often used in addition to traditional chemotherapy. If targeted therapy includes monoclonal antibodies, costs can escalate exponentially. Pharmacoeconomic studies looking at health care systems have demonstrated that the cost per life-year gained with biologics is relatively high, compared with other interventions [133–136]. For example, multidrug colorectal cancer treatment regimens containing bevacizumab or cetuximab cost up to $30,790 for eight-weeks of treatment, compared with $63 for an eight week regimen of 5-FL, the standard treatment until the mid-1990s [137, 138]. The Cost of Care Task Force of The American Society of Clinical Oncology recently released a guidance statement emphasizing the importance of physician-patient discussion regarding the cost of care and the creation of decision-making tools to allow patients to make informed and educated decisions about their treatment [139].

## 7. Conclusions

Advances in rectal cancer therapy remain stagnant despite new existing cytotoxic and targeted agents in the treatment of metastatic colorectal cancer. Traditionally, agents that have been incorporated into rectal cancer trials are those that have demonstrated promise in advanced and locally advanced colorectal cancer. However, to date, the role of cytotoxic agents as radiation sensitizers (i.e., oxaliplatin) have not demonstrated improved efficacy versus the standard of care, fluorouracil. Similarly, targeted agents have a definitive role in the treatment of metastatic colorectal cancer. Yet, its role in the treatment of rectal cancer as a radiation sensitizer has yet to be determined. In short, therapeutic developments in rectal cancer may lag behind because rectal carcinoma is not a commonly pursued area of pharmaceutical development. Furthermore, whether pCR is the optimal endpoint remains controversial versus the more traditional outcome of DFS or OS. Retrospective analyses have suggested that pCR is a potential surrogate for DFS or OS [71, 72]. These findings must be validated prospectively in a phase III trial.

Targeted agents continue to be evaluated in the phase I/II setting with promising potential but have yet to be proven to be superior to the standard of care, fluorouracil-based radiotherapy. Currently, the use of targeted agents is best suited in the setting of a clinical trial. Future developments in rectal cancer should focus on the identification of molecular factors that have prognostic and predictive significance to improve treatment outcomes. Research efforts focusing on additional biomarkers will refine our ability to use these agents specifically in patient populations that derive a meaningful benefit. With concerns about health care costs, it is also critical to create a pharmacoeconomic framework guiding the clinical use of these agents. The introduction of new therapeutic agents and the discovery and validation of prognostic and predictive markers along with new screening tools will enable oncologists to tailor patient-specific chemotherapy by maximizing drug efficacy and minimizing adverse and possibly severe side effects.

## Figures and Tables

**Table 1:** Pivotal randomized clinical trials of bevacizumab in mCRC.

Author	Phase	Study design (regimen)	*N* +Bev/−	Results	Comment	Reference
1st line						
Hurwitz	III	IFL ± bevacizumab	402/411	20.3 versus 15.6 mo OS	RR,PFS,OS benefit	[9]
Scappaticci	II	5FU/LV ± bevacizumab	104/105	9.2 versus 5.5 mo PFS	No difference in OS, IFL was included in control group	[14]
Czito	III	IFL ± bevacizumab	249/251	17.9 versus 14.6 mo OS	RR,PFS,OS benefit	[15]
Saltz	III	CAPOX or FOLFOX ± bevacizumab	699/701	9.4 versus 8.0 mo PFS	No difference in OS	[16]
2nd line						
Giantonio	III	FOLFOX ± bevacizumab	286/291	12.9 versus 10.8 mo OS	RR,PFS,OS benefit, No benefit in Bev alone group	[17]

RR: Response rate, PFS: Progression-free survival, OS: Overall survival.

**Table 2:** Pivotal randomized clinical trials of cetuximab in mCRC.

Study	Phase	Study design(regimen)	*N* +Cet/−	Results	Comment	Reference
1st line						
CRYSTAL	III	FOLFIRI ± cetuximab	599/599	No difference in OS	PFS,OS benefit in KRAS-WT	[23]
OPUS	II	FOLFOX ± cetuximab	169/168	46 versus 36% RR	No difference in OS	[24]
CAIRO-2	III	CAPOX + beva ± cetuximab	377/378	9.4 versus 10.7 mo PFS		[25]
COIN	III	CAPOX or FOLFOX ± cetuximab	815/815	17 versus 17.9 mo OS	No difference in PFS,OS in KRAS-WT	[26]
NORDIC VII	III	FLOX ± cetuximab	194/185	19.7 versus 20.4 mo OS	No difference in PFS,OS in KRAS-WT	[27]
2nd line						
BOND	II	Irinotecan ± cetuximab	218/111	22.9 versus 10.8% PR		[10]
CO.17	III	Cetuximab versus supportive care	287/285	6.1 versus 4.6 mo OS	9.5 versus 4.8 mo OS in KRAS-WT	[11, 28]

RR: Response rate, PFS: Progression-free survival, OS: Overall survival. KRAS-WT: KRAS wild type.

**Table 3:** Clinical trials of bevacizumab in preoperative treatment of rectal cancer.

Author	Phase	Study design(regimen)	*N*	RT dose (Gy)	pCR rate	Major wound Cx	Reference
Machiels	I	Cape + oxali + bevacizumab	11	50.4	2/11(18%)	NS	[45]
Willett	II	5FU + bevacizumab	32	50.4	5/32(16%)	1/32(3%)	[29]
Rodel	II	5FU + bevacizumab	35	50.4	10/35(29%)	3/20(15%)	[46]
Crane	II	Capecitabine + bevacizumab	25	50.4	8/25(32%)	3/25(12%)	[47]
Mourad	II	Xelox + beva then cape + beva + RT	47	50.4	16/47(36%)	11/47(24%)	[48]

RT: Radiotherapy, pCR: Pathological complete response.

**Table 4:** Clinical trials of cetuximab in preoperative treatment of rectal cancer.

Author	Phase	Study design(regimen)	N	RT dose (Gy)	pCR rate	Diarrhea (≥ G 3)	Reference
Kuo	I	5FU+ cetuximab	20	50.4	2/17(12%)	2/20(10%)	[67]
Bertolini	I	Caplri + cetuximab	20	50.4	5/20(25%)	2/10(20%)	[68]
Velenik	I/II	Capecitabine + cetuximab	40	45	2/40(5%)	6/40(15%)	[69]
Horisberger	I/II	CAPOX + cetuximab	48	50.4	4/48(8%)	9/48(19%)	[70]
Chang	II	Cetuximab then 5FU + cetux	40	50.4	3/40(7.5%)	3/40(7.5%)	[71]
Rodel	II	Capecitabine + cetuximab	37	45	3/37(8.1%)	4/37(11%)	[72]
Oncofacts.com	II	Caplri + cetuximab	50	50.4	4/50(8%)	15/50(30%)	[44]

RT:Radiotherapy, pCR: Pathological complete response.
